# CD163/CD63+ Monocyte-Derived DC Profiled in Tissue by Multi-Antigen Analysis (MAA) Discriminate Chronic Eczema and Psoriasis

**DOI:** 10.3390/ijms26189077

**Published:** 2025-09-18

**Authors:** Sabrina Windorfer, Michael Kirr, Waltraud Fröhlich, Bianca Plosnita, Christian Ostalecki, Carola Berking, Michael Sticherling, Andreas S. Baur

**Affiliations:** 1 Bezirkskrankenhaus Passau, Fachklinik für Psychiatrie, Psychotherapie und Psychsomatik, 94032 Passau, Germany; sabrina.windorfer@gmail.com; 2 Hautklinik, Uniklinikum Erlangen, Friedrich-Alexander-Universität Erlangen-Nürnberg, 91054 Erlangen, Germany; kirr@students.uni-marburg.de (M.K.); waltraud.froehlich@uk-erlangen.de (W.F.); c.ostalecki@gmail.com (C.O.); carola.berking@uk-erlangen.de (C.B.); michael.sticherling@uk-erlangen.de (M.S.); 3 Deutsches Zentrum Immuntherapie (DZI), Ulmenweg 18, 91054 Erlangen, Germany; 4 TissueGnostics GmbH, 1020 Vienna, Austria; bianca.plosnita@tissuegnostics.com

**Keywords:** psoriasis, eczema, atopic dermatitis, inflammatory dendritic cells, DC, multiplex imaging, multi-antigen analysis, DC3 cells, Langerhans cells

## Abstract

Psoriasis (Pso) is a chronic inflammatory skin disease with a genetic predisposition and an assumed autoimmune pathomechanism. Autoantigens, dendritic cells (DCs), and the TNF/IL23/Il17 axis are seemingly the main drivers of this process. However, the difference to other DC-driven immune processes, like in eczematic skin, is insufficiently understood. Multi-antigen analysis (MAA) allows the staining of tissue with 100 antigens and more and provides a deeper insight into pathological processes, using advanced imaging analysis and quantification of topographical allocated processes. Here we used this technology to assess and compare the skin immune infiltrations in Pso, chronic eczema, and healthy controls. Tissue samples from both skin diseases (*n* = 30) were stained for 63 antigens, including 45 immune markers, and cells were analysed and quantified in both epidermis and dermis. The presence of different types of monocyte-derived DC in the epidermis was the most notable distinction between both skin diseases. While in Pso a monocyte-derived DC (CD14+CD1a+CD11c+) predominated, possibly a Langerhans cell (LC)-like DC, eczema displayed a marker combination of a seemingly more differentiated monocyte-derived DC (CD14+CD63+CD163+), potentially a DC3 cell type.

## 1. Introduction

Psoriasis (Pso) is a common chronic inflammatory skin disorder involving dysregulated innate and adaptive immune responses [1,2,3,4,5]. Affecting 2–3% of the global population, this autoimmune disease is primarily driven by a Th17 T-cell response and associated IL-17 activation. Despite being one of the most studied autoimmune skin disorders, the variety of its clinical manifestations and therapy responses remains puzzling, suggesting that our understanding of its precise immune pathomechanism is still incomplete [6].

Conversely, the clinical appearance of eczema is more uniform and characterised by a strong Th2 component involving IL-4 and IL-13 over-production [5,7,8]. This is underscored by the great clinical success of antibodies blocking the receptors for these cytokines (IL-4Rα; IL-13Rα1) [9]. While both diseases involve inflammation and T-cells, the fundamental nature of disease-driving antigens is different. In Pso, it seems a case of mistaken identity, i.e., the immune system attacks self-antigens, including the antimicrobial peptide LL37 and keratin17 [10]. Conversely, in eczema, it is a case of over-reaction, i.e., the immune system reacts excessively to external allergens and microbial antigens that breach a weak barrier.

A widely substantiated model positions autoimmune antigens and dendritic cells (DCs) as the central driving forces of psoriatic inflammation, with critical roles for plasmacytoid DCs (pDCs) and inflammatory DCs (iDCs) [11,12,13,14]. In this cascade, autoantigens like the antimicrobial peptide cathelicidin (LL37) complex with self-DNA and act as a key activator of pDCs [15]. These activated pDCs, alongside keratinocytes and recruited iDCs, then produce IFN-α, IFN-β, TNF, and IL-23 [11,16]. IL-23 is pivotal, stimulating the generation and activation of IL-17-producing T helper cells (Th17). This ultimately leads to the pathological hyperproliferation of basal keratinocytes and a downregulation of their differentiation differentiation [17]. Conversely, in eczema, conventional DCs (cDCs) are known to induce a Th2-cell-associated immune response, resulting in T-cell secretion of IL-4, IL-5, IL-9, and IL-13. However, the specific DC subset responsible for initiating this Th2 response remains unclear, not least because these DCs themselves do not produce the polarising cytokine IL-4 [18].

The immunological landscape has been further complicated by the discovery that skin-resident Langerhans cells (LCs) can originate from monocytes during inflammatory states, challenging their traditional classification [19,20]. Additionally, inflammatory dendritic epidermal cells (IDECs) have been identified as prominent players, particularly in eczema and other inflammatory skin diseases [21,22]. In summary, the inflamed epidermis contains at least three distinct types of DCs: traditional LCs, monocyte-derived LC-like cells, and IDECs [22].

In view of these complex and sometimes conflicting findings, we reasoned that a multi-antigen analysis (MAA) approach to characterise and compare the immune infiltrates in Th1/Th17-(Pso)- and Th2-(eczema)-dominated environments could provide new insights. By quantitatively mapping topographically allocated DC populations, we successfully distinguished two dominant monocyte-derived DC subtypes that discriminate Pso from eczema.

## 2. Results

### 2.1. CD14+ and CD1a+ Cells Dominate in the Epidermis of Chronic Eczema

For our MAA technology-based study, we initially screened over 500 different antibodies and selected those that gave a strong and specific staining pattern on psoriatic and eczema tissue. A specific signal had a high signal-to-noise ratio, was localised to expected structures, and was reproducible. Specificity was confirmed by its disappearance in controls (e.g., healthy skin), correlation with the target’s known localisation, and intensity changes with antibody titration.

This screening process resulted in a selection of 63 markers, namely 46 antibodies for the characterisation of immune cells (mostly surface markers), 8 antibodies detecting structural proteins, and 9 antibodies detecting proteases and their substrates (listed in Materials and Methods). This enabled us to quantify immune cell subpopulations in immunologically distinct areas of the skin, the dermis, and the epidermis. These cells were quantified by the software Strataquest (Version 6), as described recently [23].

Using this antibody panel, we analysed a total of 35 tissue samples, including 3 clinically different Pso subtypes (psoriasis vulgaris (*n* = 15), inverse psoriasis (*n* = 5), and psoriasis guttate (*n* = 2)) and compared them with tissue samples of chronic (*n* = 7) and subacute (*n* = 2) eczema. For the statistical analysis, the sub-entities of each disease were grouped together, because we did not see significant differences between these subtypes in a retrospective evaluation. The merged subgroups are referred to as “Pso” and “eczema”. Samples from five healthy individuals served as controls.

**Figure 1:**
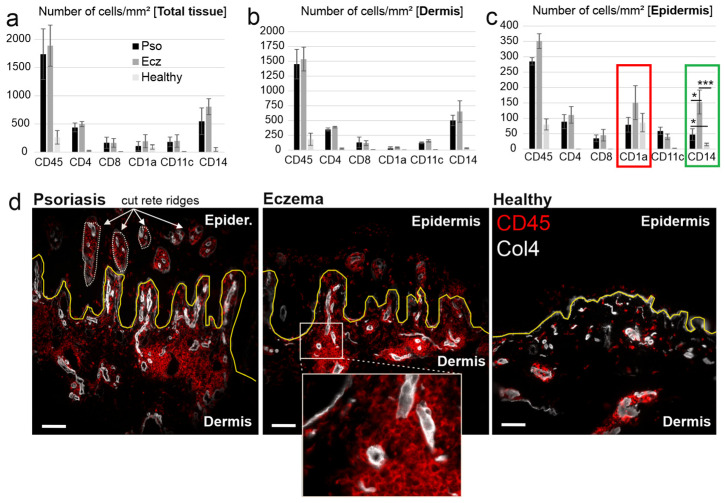
Pso and eczema differ in the number of CD1a+ and CD14+ cells in the epidermis. (**a**–**c**) Comparable numbers of immune cells in Pso and eczema in total skin (total tissue). Skin sections of Pso (Pso), chronic eczema (Ecz), and control tissue (Healthy) were subjected to MAA analysis and the number of immunoreactive cells per square millimetre in total tissue (**a**), the dermis (**b**), and the epidermis (**c**) were quantified using imaging software (Strataquest), as previously described [23,24]. The red and green boxes in (**c**) depict the increase in CD1a+ and CD14+ cells respectively in eczema over Pso. (**d**) Representative images showing an overlay of CD45+ cells (red) and blood vessels (collagen type IV, white). Collagen type IV was further used to mark the basal membrane ((BM), yellow line). White arrows and dotted lines depict cut rete ridges in psoriatic skin (see also text). The insert was added to demonstrate the resolution of the images. Data are means ± SEM from *n* patients (Pso: *n* = 19, Ecz: *n* = 9, healthy: *n* = 5). Statistical significance was assessed based on the *p* value (* *p* < 0.05, *** *p* < 0.001), and deter-mined using two-way ANOVA and Tukey’s test. Note: Non-significant results were not specifically indicated. Scale bars = 200 µm.

In order to obtain an overview on the expected increase of principle subtypes of immune cells (expressed in numbers of cells/mm^2^ of tissue, Figure 1), we analysed the following markers in our MAA: CD45 (pan leukocytes), CD4, CD8 (T cells), CD11c, CD1a (DC), and CD14 for monocytes. The results showed, that in both diseases, CD45+ immune cells increased about 6-fold in total tissue (epidermis and dermis) over controls, namely from ~260 cells/mm^2^ in controls to ~1440 cells/mm^2^ in Pso and ~1610 cells/mm^2^ in eczema (Figure 1a and representative images in Figure 1d). Among immune cell subtypes, CD14+ and CD4+ cells increased the most in both diseases, namely 10–26-fold from ~30–50 cells/mm^2^ in controls to ~480–800 cells/mm^2^ (Figure 1a). There was a trend towards higher numbers in eczema compared with Pso, particularly in CD14+ cells. Also, CD11c+ cells increased in both diseases from ~5 cells/mm^2^ in controls to ~200 cells/mm^2^ (Figure 1a). While this was a 40-fold increase, the overall numbers were about 3–4 times lower when compared with CD14+ cells. Conversely, CD1a+ cells (presumably LC) did not increase much in both diseases, only ~2.5-fold in eczema, namely from ~80 cells/mm^2^ to ~200 cells/mm^2^. Overall, there was a trend towards higher CD14+ and CD1a+ cells in eczema compared with Pso, but no significant difference was observed (Figure 1a). Please note, non-significant differences were not specifically indicated in figures.

**Figure 2:**
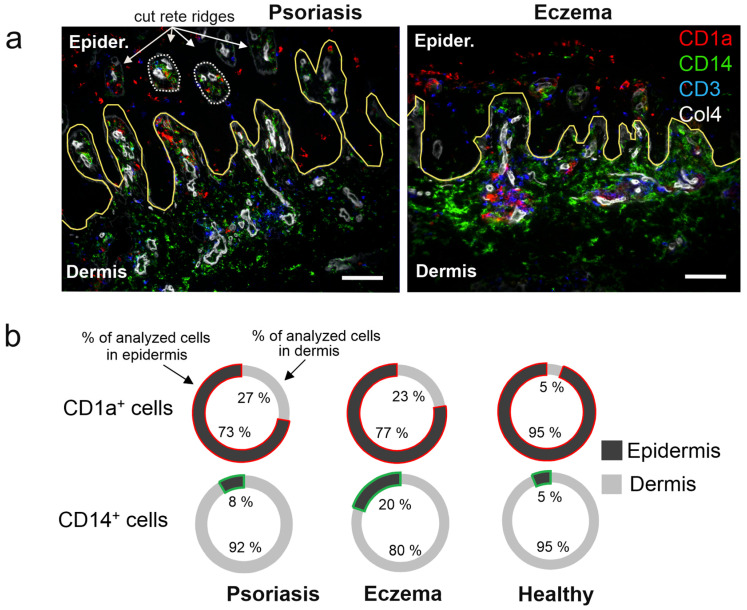
In Eczema, CD14+ cells are recruited to the epidermis at a higher proportion/number. (**a**) Representative images showing markers for CD1a (red), CD14c (green), CD3 (blue), collagen type IV (Col4, white), and basal membrane (BM) in yellow. White arrows and dotted lines depict cut rete ridges. (**b**) Pie charts showing the relative percentage/proportion of CD1a+ and CD14+ immune cells in Pso, eczema, and healthy tissue within epidermis and dermis. Numbers in the pie chart are based on cell numbers per square mm tissue of the respective tissue.

Next, immune cell presence was quantified separately in the dermis and epidermis. In the dermis, no significant differences between Pso and eczema were noted (Figure 1b) and the results were similar to those shown in Figure 1a. Conversely, in the epidermis, eczema revealed a 3.3 respective 10-fold increase (≈150 cells/mm^2^) over Pso (≈45 cells/mm^2^) and healthy controls (≈15 cells/mm^2^) (Figure 1c, green box). In addition, CD1a+ cells were twice as frequent in eczema compared with Pso and healthy controls (~150 cells/mm^2^ in eczema vs. ~80 cells/mm^2^ in Pso vs. ~85 cells/mm^2^ in healthy) (Figure 1c, red box). Please note, psoriatic skin sections typically harbour cut rete ridges, belonging to the dermis (see white arrows in Figure 1d and Figure 2a). Cells in these areas were excluded from the analysis in Figure 1c and Figure 2b. In summary, the presence of CD14+ cells, and to some degree CD1a+ cells, seemed characteristic for eczema compared with Pso.

### 2.2. High Recruitment of CD14+ Cells to the Eczema Epidermis

The topographical distribution of the main cell types (CD1a, CD3, CD14) in the dermis and epidermis, and the accumulation of CD14+ cells in particular, is shown in a representative overlay image (Figure 2a). To detail these results, we compared the distribution percentage (cells per mm^2^) of CD1a+ and CD14+ cells in the epidermis versus dermis for each disease and controls. The presence of CD1a+ cells (presumably LC) in the epidermis and dermis was quite similar in Pso and eczema (epidermis/dermis: 73/27% in Pso vs. 77/23% in eczema) (Figure 2b, upper circles). In healthy skin, almost all CD1a+ cells were located in the epidermis (epidermis/dermis:95/5%). On the other hand, we noted a pronounced difference in CD14+ cell distribution (Figure 2b, lower circles). In Pso, almost all CD14+ cells were located in the dermis (dermis vs. epidermis: 92% vs. 8%), whereas in eczema a larger proportion was found in the epidermis (dermis vs. epidermis: 80% vs. 20%). Together with the absolute cell numbers shown in Figure 1, this implied that in eczema, monocytes and/or CD14+ cells were recruited to the epidermis at a higher proportion/number.

### 2.3. DC Expressing CD63 and CD163 Are Predominant in Eczema

Since numbers of CD1a+ and CD14+ cells revealed notable differences in Pso and eczema, we decided to characterise these cells in more detail by including additional markers. In the course of our analysis, we noted an increased presence of immune cells harbouring CD11c, CD63, and CD163. CD163 had been found on monocyte-derived DC3 cells priming CD8 cells [25], whereas CD63 was found on immature monocyte-derived DC [26]. Furthermore, CD63 is a marker for exosomes, which are commonly secreted by monocytes and DC [14]. Hence, we determined the relative presence of immune cells with different triple marker combinations of CD1a, CD14, CD11c, CD63, and CD163.

**Figure 3:**
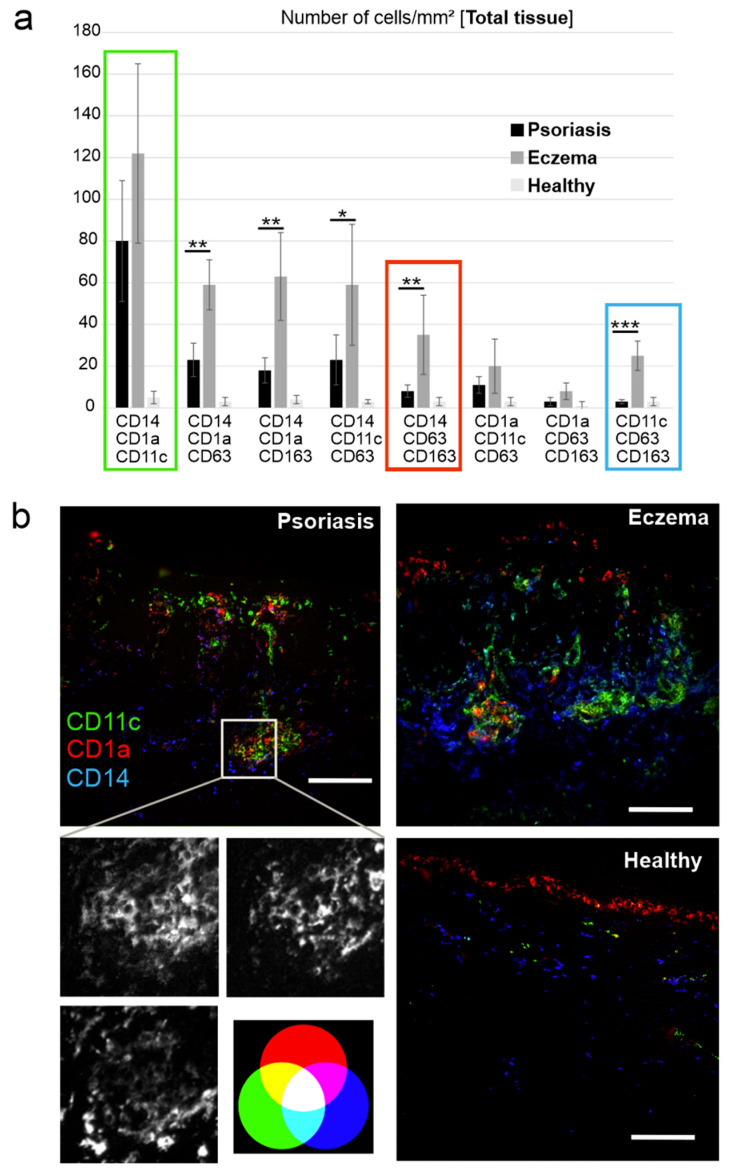
CD14+ cells harbouring DC markers (CD11c, CD1a) and CD63 and/or CD163 are representative for eczema. (**a**) Quantitative analysis of the different triple positive immune subtypes in Pso, eczema, and healthy tissue by MAA, characterised by indicated markers. (**b**) Representative co-localisation images for CD1a (red), CD11c (green), CD14 (blue), seen in Pso and eczema (here shown in Pso.). Scale bars = 200 µm and 20 µm (close-up image); data are means ± SEM from *n* patients (Pso: *n* = 19, Ecz: *n* = 9, healthy: *n* = 5). Statistical significance was assessed based on the *p* value (* *p* < 0.05, ** *p* < 0.01, *** *p* < 0.001), and determined using two-way ANOVA and Tukey’s test. Note: Non-significant results were not specifically indicated. Scale bars = 200 µm.

The most prevalent triple combination (CD14, CD1a, CD11c) was present in both Pso (≈80 cells/mm^2^) and chronic eczema (≈120 cells/mm2) (Figure 3a, green box; Figure 3b). Triple marker combinations that included CD63 and/or CD163 were each somewhat less frequent in number; however, together they were the most frequent DC populations in eczema (Figure 3a, grey bars), and significantly more prevalent than in Pso (Figure 3a, black bars). For example, the marker combination CD14, CD63, and CD163 was 4.4-fold higher in eczema compared with Pso (~35 cells/mm^2^ vs. ~8 cells/mm^2^, Figure 3a, red box), and the marker combination CD11c, CD63, and CD163 was 8.4 fold higher (~25 cells/mm^2^ vs. 3 cells/mm^2^) (Figure 3a, blue box). Notably, this latter triple combination was almost absent in Pso. In healthy tissue, hardly any of these marker combinations were observed. In summary, CD14+ positive cells harbouring DC markers (CD11c, CD1a) in combination with CD63 and/or CD163 were far more present in eczema compared with Pso.

### 2.4. CD11c+/CD63+/CD163+ Cells Discriminate Pso from Eczema

**Figure 4:**
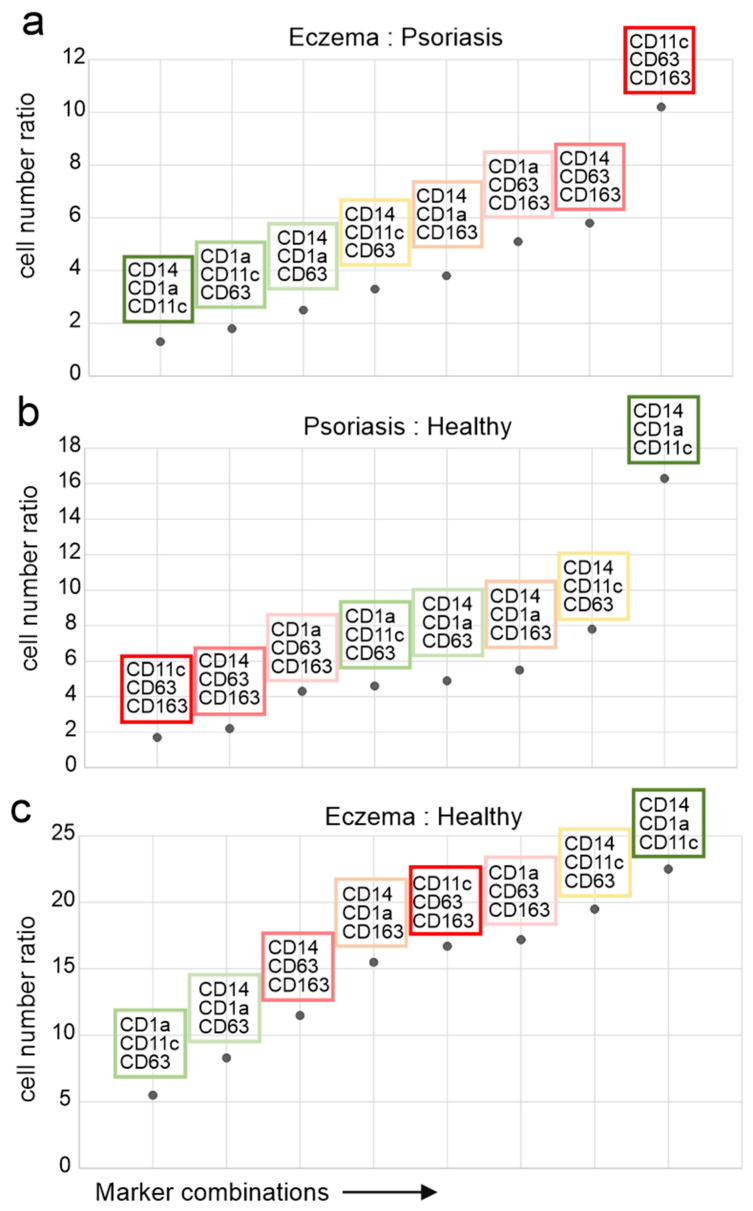
CD11c/CD63/CD163+ DC discriminates eczema from Pso. Pairwise comparison of triple marker combinations expressed as ratio of cell numbers of one condition over the other. (**a**) Eczema over Pso, (**b**) Pso over healthy skin, and (**c**) eczema over healthy skin. The vertical numbers depict the ratio or fold increase. The marker combination discriminating two groups the best is seen in the upper right quadrant. Each marker combination has a representative colour frame.

The results of our analysis led to the question, which of these triple positive cell subpopulations would distinguish two given skin conditions, most importantly Pso and eczema. To this end, we calculated the relative presence of a given triple marker combination (cells/mm^2^), and expressed the difference between two skin conditions by a cell number ratio. For example, a marker combination present in similar numbers in both conditions would have a cell number ratio of 1:1, or 1. By this approach, we aimed to identify marker combinations that were most distinctive for a given pair of skin conditions. These ratios are displayed in three scatter plots, each showing a comparison between two skin conditions (Figure 4). For eczema and Pso, the appearance of CD63 and/or CD163 in combination with CD14 and/or CD1a and/or CD11c in eczema was most discriminatory. Among those, the combination CD11c, CD63, and CD163 showed the highest ratio of 10:1, or 10 (Figure 4a, bright red box). Supporting this conclusion, a marker combination not containing CD63 and/or CD163 best discriminated Pso from healthy skin, namely CD14, CD1a, and CD11c (Ratio 16:1; Figure 4b, dark green box). The same marker combination was also best in discriminating eczema from healthy skin (23:1; Figure 4c), as this marker combination was equally present in Pso and eczema (Figure 4a, ratio 1:1). However, marker combinations that included CD63 and/or CD163 were comparably present (ratios 15–20:1; Figure 4c), making these marker combinations representative for eczema but not for Pso. In summary, the appearance of CD63 and/or CD163 on CD14+ and/or CD1a/CD11c+ cells were most characteristic and distinctive for eczema skin, whereas the same cells were lacking CD63/CD163 in psoriatic skin.

## 3. Discussion

In this multi-antigen analysis (MAA) on Pso and eczema skin tissue samples, we analysed 63 antigens on skin immune cells of Pso and eczema in a similar fashion as previously demonstrated [23,27,28]. We came to at least two conclusions. First, the high prevalence of CD14 in conjunction with classical DC markers suggests that the main fraction of DC found in both diseases are derived from monocytes. Second, the marker expression on these cells differed in both diseases. While Pso cells with DC markers maintained a CD14+, potentially LC-like phenotype (CD14+CD1a+CD11c+) [29], cells in eczema displayed a marker combination that included CD163 and/or CD63, hinting at a more differentiated DC that may constitute a DC3 subtype, as previously suggested [30,31].

Numerous publications have characterised DC in psoriatic skin (Pso) and eczema/atopic dermatitis (AD). For many years this has been performed by staining formaldehyde-fixed paraffin-embedded tissue (FFPE) with few antibodies. More recently more sophisticated approaches have been applied, for example by using RNA sequencing of sorted cells from tissue samples [30]. The latter approach gave a more differentiated but also more complex picture of DC in chronic inflammatory diseases. A similar MAA-based study on Pso and AD skin tissue was recently published, which included Olink proteomics and single-cell phenotyping [32]. Only modest disease-specific differences were observed in T-cell populations, but stronger differences were noted in macrophage and DC populations. While some findings were similar to ours, like the concentrations of the main immune cell populations in Pso and AD, the authors found large DC1 and DC2 cell populations in Pso and CCR4+CD206+CD123+ DC in AD. The latter are associated with antiviral and tolerogenic functions. Like in the presented study, they identified a large proportion of CD14+ cells; however, the authors interpreted these as mainly anti-inflammatory macrophages. In our study we did not detect CD68, a robust macrophage-specific marker, on CD14+ cells. Therefore, and because of their co-expression of typical DC markers (CD1a, CD11c), we interpreted these cells as monocyte-derived DCs. This seems to be in agreement with previous findings [33].

In Pso, the CD14+ monocyte-derived cells had a more uniform appearance, which included the DC marker combination CD1a and CD11c (Figure 3a and Figure 4). Conversely, in eczema these cells additionally expressed CD63 and CD163 in various combinations with CD1a and CD11c. Supporting this conclusion, the marker combinations CD11c, CD63, CD163 and CD14, CD63, CD163 were almost exclusively found in eczema (Figure 3a). In addition, CD14+ cells were prominent in both the dermis and the epidermis of eczema (Figure 1c,d and Figure 2b). Taken together in a speculative model, in eczema, monocytes are recruited through the dermis to the site of inflammation in the epidermis, where they develop into inflammatory and, eventually, mature DCs. Potentially these cells are a DC3 type/subtype as recently described [25,30]. Notably, Nakamizo et al. [30] found this cell population in Pso, while we detected these cells at higher concentrations in eczema. The difference could be due to the different analysis methods that were applied.

**Figure 5:**
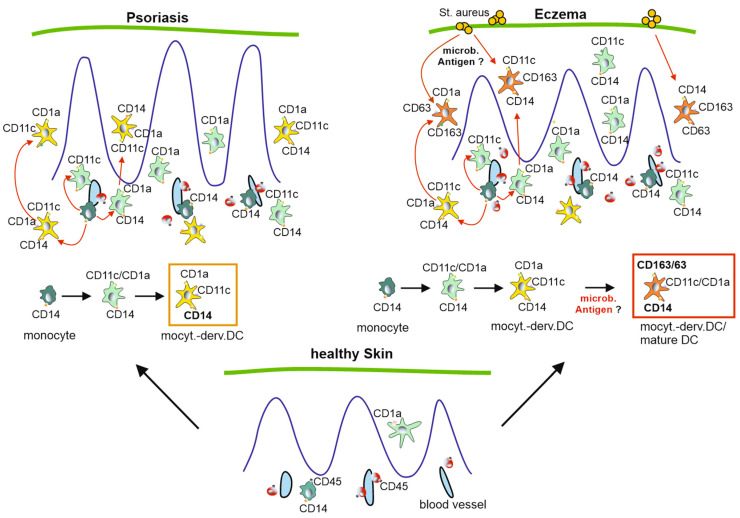
Speculative model on the divergent development of monocyte-derived DCs in psoriasis and eczema. In both diseases the dominant DC populations seem to derive from monocytes that are recruited to the dermis and subsequently to the epidermis from the bloodstream (bottom, healthy skin). In the course of this migration, the cells develop into CD1a/CD11c/CD14+ cells (depicted in yellow) in both diseases (arrows), potentially a LC-like cell type. In Pso these cells predominate, potentially reaching their final differentiation state. Conversely, in eczema, these DCs further differentiate into a more mature DC co-expressing CD63 and or CD163 (depicted in orange), potentially due to the presence of microbial antigens and exotoxins (likely from staph aureus). These cells may constitute a DC3 cell type.

The differentiation of DC in eczema is potentially induced by bacterial-derived antigens (likely from Staphylococcus aureus), eventually leading to the expression of CD163 and CD63 (Figure 5), although the latter marker has been described as being expressed on immature DCs [26]. In Pso, most of these cells seemingly stop differentiating at a level that includes the markers CD14, CD1a, and CD11c in different combinations (Figure 4a,b and Figure 5). In fact, this is not unexpected as Pso constitutes a sterile skin disease/inflammatory process with a genetic predisposition, whereas AD is typically driven by the superinfection of microbes (mainly Staph. aureus). Taken together, microbial antigens may stimulate a differentiation process that could explain the distinct appearance of monocyte-derived inflammatory DCs in eczema (Figure 5).

Monocytes are attracted to the skin by chemokines, potentially released by resident skin cells and/or mature DCs [34,35]. As we have demonstrated previously in mouse skin, extracellular vesicles from mature DCs, but not from immature DCs, have the capacity to differentiate monocytes into immature DCs [24]. Upon further differentiation/maturation, for example following the uptake of bacterial antigen, these cells would release EV with the capacity to recruit and differentiate more monocytes, leading to a perpetuation of this mechanism as long as there is a maturation stimulus present, e.g., bacterial antigen. Ultimately, this mechanism would lead to chronic inflammation. Obviously, the situation in Pso is different, as microbial antigens are not driving the inflammatory process. Hence, we may speculate, that in Pso, DCs and/or additional cells are stimulated by autoantigens like the one described by Lande et al. [15], leading to a differentiation of monocytes into immature inflammatory DCs. This process may terminate at an immature level, perhaps due to the lack of classical maturation stimuli. Certainly, additional work is necessary to substantiate or exclude this hypothetical mechanism.

## 4. Materials and Methods

### 4.1. Tissue Samples

We collected tissue samples from 35 patients treated in the Department of Dermatology at the University Hospital Erlangen. The cohort included 15 samples from patients with Pso vulgaris, 5 with inverse Pso, 2 with Pso guttata, 7 with chronic eczema, 2 with subacute eczema, and 5 healthy skin controls. Each lesional skin sample (excluding healthy skin) was obtained from distinct individuals and specifically from affected skin areas. The collection of samples was conducted following approval by the local ethics committee. To ensure accurate diagnoses, we evaluated both the clinical presentation and histopathological features of the samples. Lesional skin samples from patients with Pso and eczema were obtained from outpatients at the Hautklinik, while healthy skin samples were sourced from dermatological surgical procedures. Clinical diagnoses were established based on a combination of clinical examination and histopathological analysis.

### 4.2. MAA Sample Preparation

After receiving the samples by skin punch surgery, they were transferred to the research department in a 0.9% sodium chloride solution. Without undue delay, the samples were frozen at −80 °C in O.C.T. compound (Sakura Finetek, Torrance, CA, USA). Using a cryotome, 5 μm sections were prepared and fixed in acetone on microscope slides for 10 s, air dried for 10 min, and finally stored at −80 °C.

### 4.3. MAA Data Generation

Multi-antigen analysis (MAA) allows to locate and visualise numerous different structures marked by specific antibodies in a single tissue sample, using repetitive cycles of immunostaining and bleaching [36]. A single tissue slide was placed under a fluorescence microscope of a staining robot. During the robotic process FITC (fluorescein isothiocyanate)-, PE (phycoerythrin)- or AF488 (Alexa fluor)-labelled antibodies were incubated with the tissue, followed by washing with PBS at 20 °C and a bleaching process. The latter led to the inactivation of the fluorochrome-labelled antibodies before a new cycle ensued. To obtain the fluorescence images, an inverted wide-field fluorescence microscope (DM IRE2; ×20 air lens; numerical aperture 0.7; Leica, Wetzlar, Germany) with a cooled CCD camera (KX4; Apogee Imaging Systems, Roseville, CA, USA) was used. All system components were coordinated by software developed by MelTec GmbH & Co KG (Magdeburg, Germany). To guarantee high quality images, each obtained image was pixel-wise aligned and corrected for illumination faults using flat-field correction. For this process, the corresponding phase-contrast images were used. This arrangement reached a resolution of ±1 pixel. Superimposed images composed an *n* epitope presence in relation to each pixel (approximately 900 nm × 900 nm area) of a visual field (2048 × 2048 pixels).

### 4.4. MAA Antibody Library

The following antibodies were used to immunohistochemically detect the antigens in the tissue samples: anti-ADAM10-PE (SHM14, Biolegend, 352704, Taipei City, Taiwan), anti-ADAM17-FITC (111633, R&D Systems, Minneapolis, MN, USA, FAB9301F), E-Cadherin-FITC (36/E-Cadherin, BD, 612131), anti-beta-Catenin-FITC (12F7, Novus, Singapore, NBP1-54467F), anti-CD1a-PE (NA1/34, Dako, R7189), anti-CD3-PE (UCHT1, ImmunoTools, Friesoythe, Germany, 21620034S), anti-CD4-PE (OKT-4, ImmunoTools, 21850044S), anti-CD6-PE (HI210, ImmunoTools, 21810064S), anti-CD8-PE (HIT8a, ImmunoTools, 21810084S), anti-CD9-PE (HI9a, ImmunoTools, 21810094S), anti-CD11a-PE (HI111, ImmunoTools, 21810114S), anti-CD11c-PE (BU15, ImmunoTools, 21487114S), anti-CD14-PE (18D11, ImmunoTools, 21620144S), anti-CD16-PE (3G8, Beckmann Coulter, A07766), anti-CD19-PE (HIB19 Klon LT19, ImmunoTools, 21270194S), anti-CD20-FITC (REA780, Miltenyi Biotec, Bergisch Gladbach, Germany), anti-CD24-FITC (SN3, ImmunoTools, 21270243S), anti-CD25-PE (HI25a, ImmunoTools, 21810254S), anti-CD29-FITC (HI29a, ImmunoTools, 21810293S), anti-CD36-FITC (TR9, ImmunoTools, 21270363S), anti-CD38-PE (HIT2, ImmunoTools, 21270384S), anti-CD40-PE (HI40a, ImmunoTools, 21270404S), anti-CD44-PE (IM7, ImmunoTools, 21850444S), anti-CD45-PE (HI30, ImmunoTools. 21810454S), anti-CD45RA-PE (HI100, ImmunoTools, 21819454S), anti-CD45RO-FITC (UCHL1, ImmunoTools, 21336453S), anti-CD52-FITC (HI186, ImmunoTools, 21270523S), anti-CD54-FITC (1H4, ImmunoTools, 21279543S), anti-CD55-PE (HI55a, ImmunoTools, 21810554S), anti-CD56-PE (B-A19, ImmunoTools, 21810564S), anti-CD62P-PE (HI62P, ImmunoTools, 21270624S), anti-CD63-FITC (MEM-259, BioLegend, 312004), anti-CD68-FITC (KP1, Dako, F 7135), anti-CD69-PE (IT8G1, ImmunoTools, 21459694), anti-CD71-FITC (M-A712, BD Pharmingen, 555536), anti-CD95-PE (LT95, ImmunoTools, 21278954S), anti-CD107a-FITC (H4A3, BD Pharmingen, 555800), anti-CD123-FITC (7G3, BD Pharmingen, 558663), anti-CD138-FITC (DL-101, BioLegend, 352304), anti-CD163-PE (GHI/61, BioLegend, 333606), anti-CD206-FITC (DCN228, Miltenyi Biotec, 130-095-131), anti-CD209-FITC (DCN47.5, Miltenyi Biotec, 130-092-873), anti-CD271-FITC (ME20.4-1.H4, Miltenyi Biotec, 130-091-917), anti-Collagen type IV-FITC (5K134, US Biological, C7510-50R1), anti-phospho-Connexin-FITC (polyclonal, Santa Cruz, CA, USA, sc-17218 FITC), anti-Cytokeratin-14-FITC (LL002, abcam, ab77684), EGFR-AF488 (AY13, BioLegend, 352908), anti-HLA-ABC-PE (W6/32, ImmunoTools, 21159034S), anti-HLA-DR-PE (HI43, ImmunoTools, 21819984S), anti-KIP1-FITC (11F10, SMA HZM, self-labelled), anti-Ki67-AF488 (Ki67 monoclonal, Biolegend, F0788), anti-L302-FITC (6A2, SMA HZM, self-labelled), anti-Melan-A-FITC (A103, Santa Cruz, sc-20032 FITC), anti-Nestin-AF488 (10C2, BioLegend, 656812), anti-Notch1-FITC (mN1A, abcam, ab80045), anti-Notch3-PE (MHN3-21, BioLegend, 345406), anti-Notch4-PE (MHN4-2, BioLegend, 349004), anti-p63-FITC (4A4, Santa Cruz, sc-8431 FITC), anti-PPARgamma-FITC (E-8, Santa Cruz, sc-7273 FITC), anti-PPB-FITC (1G6, SMA HZM, self-labelled), anti-TAP73-FITC (5G7, SMA HZM, self-labelled), anti-TNR1-PE (16803, R&D Systems, FAB225P), anti-TNFR2-PE (22235, R&D Systems, FAB226P), anti-Vimentin-FITC (V9, Santa Cruz, sc-6260 FITC), and propidium iodide (Genaxxon Bioscience, Ulm, Germany, M3181.0010). Initial runs served for the calibration to determine the best working dilutions of the chosen antibodies for the MAA. As a next step, the dilutions had to be adjusted if necessary and then re-examined by new test runs.

For isotype control runs we used the following antibodies: Mouse IgG1-PE (1F8, ImmunoTools, 21815014S), Mouse IgG2a-FITC (X5563, ImmunoTools, 21815023S), Mouse IgG2b-FITC (TG1.7, ImmunoTools, 21815033S), Mouse IgG3-FITC (PPV-07, ImmunoTools, 21275043).

### 4.5. MAA Analysis

The fluorescence images were analysed using StrataQuest Analysis Software^©^ Version 6 (TissueGnostics GmbH, Vienna, Austria). Propidium iodide (PI) staining was employed as a key method to detect cell nuclei. Utilising a predefined algorithm, the cytoplasm of each cell was predicted, generating a cellular mask. This mask enabled the superimposition of PI-stained fluorescence images with individual FITC-, PE-, and AF488-labelled antibody tissue images. This approach allowed for the simultaneous analysis of two critical parameters: (1) the exact number of cells expressing a specific antigen, as determined from the superimposed image, and (2) the expression level of the specific antigen, as observed in the antibody-stained image.

The next crucial step involved manually adjusting the thresholds for detecting cell nuclei size, cellular masks, and positive antigen expression. To minimise the risk of artifacts influencing the results, these artifacts were carefully removed. This process enabled the calculation of three key metrics: (1) the total cell density per square millimetre, (2) the density of cells expressing a specific antigen per square millimetre, and (3) the relative sum intensity of the expression of a specific antigen within a tissue section. Furthermore, the superimposition of multiple tissue images facilitated the representation and analysis of entire antigen expression patterns, taking into account their spatial arrangement. This method also allowed for the detection of cells identified as double or triple positive, providing a more accurate characterisation of cell subpopulations.

For example, the principle of additive colour mixing was demonstrated using cells triple positive for CD1a+CD11c+CD14+ (Figure 2a). In this case, cells expressing CD1a (red signal), CD11c (green signal), and CD14 (blue signal) were analysed. Overlaying the red and green signals resulted in a yellow signal, indicating double positivity for CD1a and CD11c. Similarly, triple positivity was detected by overlaying three images showing signals for CD1a, CD11c, and CD14. Here, the combination of blue and red signals produced a magenta signal, while the overlay of blue, green, and red signals resulted in a white signal, reflecting triple positivity.

This approach not only enhanced the accuracy of cell characterisation but also provided a robust framework for analysing complex patterns of antigen expression and identifying distinct cell subpopulations.

### 4.6. Statistical Analysis

GraphPad Prism (Version 8, GraphPad Software, San Diego, CA, USA) was used for heat maps and statistical analysis (one- and two-way ANOVA and Tukey’s, Bonferroni’s, and Dunnett’s test for multiple comparison tests). Data were expressed as mean ± standard error of the mean unless otherwise stated.

## Data Availability

Data are contained within this article.
